# Anticipating control over aversive stimuli is mediated by the medial prefrontal cortex: An fMRI study with healthy adults

**DOI:** 10.1002/hbm.25549

**Published:** 2021-06-09

**Authors:** Laura Maria Wade‐Bohleber, Amelie Haugg, Sabrina Huber, Jutta Ernst, Simone Grimm, Dominique Recher, Andre Richter, Erich Seifritz, Heinz Boeker, Georg Northoff

**Affiliations:** ^1^ Department of Psychiatry, Psychotherapy, and Psychosomatics University Hospital of Psychiatry Zurich Zurich Switzerland; ^2^ Psychological Institute Zurich University of Applied Sciences Zurich Switzerland; ^3^ Medical School Berlin Berlin Germany; ^4^ Department of Psychiatry Charité, Campus Benjamin Franklin Berlin Germany; ^5^ Department of Consultation‐Liaison‐Psychiatry and Psychosomatic Medicine University Hospital Zurich, University of Zurich Zurich Switzerland; ^6^ Department of Psychiatry University of Ottawa, Institute of Mental Health Research Ottawa Ontario Canada

**Keywords:** aversion, control anticipation, functional magnetic resonance imaging, medial prefrontal cortex

## Abstract

The anticipation of control over aversive events in life is relevant for our mental health. Insights on the underlying neural mechanisms remain limited. We developed a new functional magnetic resonance imaging (fMRI) task that uses auditory stimuli to explore the neural correlates of (1) the anticipation of control over aversion and (2) the processing of aversion. In a sample of 25 healthy adults, we observed increased neural activation in the medial prefrontal cortex (ventromedial prefrontal cortex and rostral anterior cingulate cortex), other brain areas relevant for reward anticipation (ventral striatum, brainstem [ventral tegmental area], midcingulate cortex), and the posterior cingulate cortex when they anticipated control over aversion compared with anticipating no control (1). The processing of aversive sounds compared to neutral sounds (2) was associated with increased neural activation in the bilateral posterior insula. Our findings provide evidence for the important role of medial prefrontal regions in control anticipation and highlight the relevance of conceiving the neural mechanisms involved within a reward‐based framework.

## Graphical abstract

The anticipation of control over aversive events in life is relevant for our mental health. In healthy adults, control anticipation over aversive sounds is associated with increased neural activation in the medial prefrontal cortex and other brain areas relevant for reward anticipation.

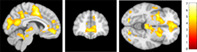

## 1 INTRODUCTION

The anticipation of aversive events serves an adaptive purpose in human life: it is a preparatory response that allows us to forecast dangers and avoid negative or harmful experiences (Andrzejewski, Greenberg, & Carlson, 2019; Butz, Sigaud, & Gérard, 2003). The perception of control is an important dimension of anticipating aversive events affecting our cognitive, emotional, and behavioral responses (Bandura, 1983; Lazarus & Folkman, 1984). The anticipation of control over future events is generally highly relevant for our mental health, a fact reflected by multiple psychological concepts and a broad array of behavioral research (cf. Skinner & Greene, 2008). Moreover, aberrant perception of control has been identified as an important factor in the pathogenesis of affective disorders (Gallagher, Bentley, & Barlow, 2014; Liu, Kleiman, Nestor, & Cheek, 2015). Most prominently, the concept of *learned helplessness*, that is, the anticipation of no control over the outcome of a situation, has been a major theme in depression research (Pryce et al., 2011). It stems from the pioneering work of Maier & Seligman, 2016 demonstrating that exposing animals to an inescapable aversive stimulation leads to a failure to escape in an analogous subsequent situation. The authors thus assumed a generalized loss of control in these animals. In 2016, Maier and Seligman altered their original theoretical formulations in light of recent findings in the neurosciences (Maier & Seligman, 2016). They postulated that passivity in reaction to an aversive stimulation actually is the “default” response and what animals and humans learn is that they can exert control over an aversive stimulation. Based on findings of mostly rodent studies, they postulated that crucial brain regions for the processing of aversive stimulation and the anticipation to control aversive stimulation are the dorsal raphe nucleus and the ventromedial prefrontal cortex (vmPFC). Hereby, the vmPFC is thought to play a crucial role in both detecting control over an aversive stimulus and inhibiting activation of the dorsal raphe nucleus. Neural projections of the dorsal raphe nucleus connect to other brain structures such as the amygdala and the dorsal periaqueductal gray, which are thought to underpin typical learned helplessness behavior.

So far, only a few studies have explored the neural correlates of the anticipation of control in the context of aversive stimulation in humans. A functional magnetic resonance imaging (fMRI) study by Kerr, McLaren, Mathy, and Nitschke (2012) showed that increased vmPFC activation is associated with the controllability of exposure to phobic stimuli, thereby providing further evidence for the implication of the vmPFC in the perception of control. In contrast, a study exploring neural activation in a fear‐conditioning task found that diminished vmPFC activity related to threat predictability and controllability (Wood et al., 2015). Another study showed that the perception of pain depends on its perceived controllability and is predicted by neural activation in the ventrolateral prefrontal cortex and the rostral anterior cingulate cortex (rACC) (Salomons, Johnstone, Backonja, Shackman, & Davidson, 2007). Moreover, a study using repetitive transcranial magnetic stimulation of the left dorsolateral prefrontal cortex in an aversive stimulation paradigm yielded inconclusive results (Taylor et al., 2014). In sum, these insights on neural correlates of the anticipation of control over an aversive stimulation in humans remain limited and are partly contradictory. In view of these contradictory findings, it is important to address that Maier and Seligman's (2016) assumptions concerning vmPFC function are mainly based on evidence in rodents. Control over an aversive stimulation in rodents needs to be differentiated from the concept of cognitive control in human research. Here, evidence suggests the differential implication of prefrontal regions. For instance, Dixon, Thiruchselvam, Todd, and Christoff (2017) proposed a composite model of regulatory cognitive functions of different parts of the frontal lobe in the context of emotion processing. They emphasize that lateral parts of the prefrontal lobe take a crucial part in appraisal and regulation whereas the rostral part of the vmPFC plays a central role in the processing of self‐relevant information. The implication of more lateral or more medial parts of the prefrontal lobe in the anticipation of control in humans remains to be clarified.

The anticipation of control over an aversive stimulation can also be conceptualized within a reward‐based framework (Ly, Wang, Bhanji, & Delgado, 2019). Such a framework stresses that conceiving control anticipation as a consequence of reinforced learning (i.e., learned associations of certain behavior with certain outcomes and the generalization of these associations to novel situations) may be too restrictive. Emotional and motivational aspects, especially concerning outcome and choice, may also have an important impact on the development of control anticipation. For instance, the nature of the outcome evoked may influence our learning. Moreover, anticipating control may reflect a basic human need or desire for control (White, 1959). However, the exact mechanisms underlying the anticipation of control over aversive stimulation are still not fully understood. In specific, the role of the reward system in these mechanisms is still debated as reward involvement is not necessary for the avoidance of aversive stimulation which can also be explained by operant reinforcement through aversion (Bouton, 2007).

Not taking into account the dimension of control, there is a consistent body of research referring to the neural basis of the anticipation and the processing of aversive stimulation. A recent meta‐analysis of the neural correlates of aversive anticipation identified a core circuit including the anterior insula, the anterior cingulate cortex (ACC), the midcingulate cortex (MCC), the amygdala, thalamus, and caudate nucleus (Andrzejewski et al., 2019). Many of these regions overlap with areas identified as being crucial for the anticipation of rewards (Gu et al., 2019; X. Liu, Hairston, Schrier, & Fan, 2011; Wilson et al., 2018). Concerning the actual processing of aversive stimulation, Hayes and Northoff (2011) showed that the most consistent findings across both human and animal studies demonstrate the implication of the amygdala, the anterior insula, the ventrolateral orbitofrontal cortex, and the rACC. Both of these meta‐analyses on the anticipation and the processing of aversive stimulation (Andrzejewski et al., 2019; Hayes & Northoff, 2011) considered studies using different sensory modalities. So far, only few studies have used auditory aversive stimuli (Bolstad et al., 2013; Carlson, Greenberg, Rubin, & Mujica‐Parodi, 2011) despite of the potential that auditory stimulation hold for experimental tasks, for example, in the context of fMRI with humans.

### 1.1 Aims and hypotheses

The main aim of this study was to explore the neural correlates of the anticipation of control over aversive auditory stimulation. Second, we explored the neural basis of the processing of aversive auditory stimulation. We developed a new fMRI task where we manipulated participants' anticipation of control, that is, their expectation of whether they had an influence on the length of different aversive sounds. We tested this fMRI task in healthy participants and investigated neural activation (1) during the anticipation of control over aversive sounds and (2) during the processing of aversive sounds. For (1) we expected to see enhanced neural activation in the vmPFC given previous evidence (mostly from rodent studies) demonstrating its implication in control anticipation (Maier & Seligman, 2016). Moreover, we hypothesized that participants of our task would experience control anticipation as rewarding. In consequence, we assumed that additional regions of the reward system would show modulated neural activation. We expected the implication of regions of the reward system that also mediate anticipatory responses, both in the context of aversion and reward (Andrzejewski et al., 2019; Gu et al., 2019; Wilson et al., 2018): the rACC, the anterior insula, the ventral striatum, and the amygdala. Finally, concerning (2) and in line with Hayes and Northoff (2011), we hypothesized that neural activation would increase in the anterior insula, the rACC, the ventrolateral orbitofrontal cortex, and the amygdala when participants processed aversive compared to neutral sounds. These regions have been most consistently implicated in the processing of aversive stimuli.

## 2 METHODS

### 2.1 Participants

Subjects were eligible for study participation if they were between 18 and 65 years of age and presented characteristics conform with MRI safety regulations (such as no pregnancy, no metallic implants, no claustrophobia, …). Exclusion criteria were any type of current mental disorder and any history of substance abuse or of a depressive or psychotic disorder. Psychiatric symptoms were assessed in a diagnostic short interview for mental disorders (mini‐DIPS [Diagnostisches Kurz‐Interview bei psychischen Störungen], Margraf, 1994). Twenty‐eight participants were recruited via a university mailing list, a local Internet platform, and the distribution of flyers in a nearby orthopedic hospital and the university campus. Three participants had to be excluded: One participant was not able to perform the task in the MRI scanner and abandoned, for one participant the logfiles of the stimuli presentation and therefore the task's timing parameters were missing, and one participant had to be excluded post hoc due to excessive head movement (>3 mm). This resulted in a final sample of 25. Mean age was 36.11 (*SD* = 13.51), education averaged 14 years (*SD* = 3.77), 17 participants were female. The ethics committee of the canton of Zurich approved the study, and all participants gave their written informed consent.

### 2.2 Procedures

Before fMRI scanning, a psychologist conducted the mini‐DIPS (diagnostic short interview for mental disorders [Diagnostisches Kurz‐Interview bei psychischen Störungen], Margraf, 1994) with the participants. Participants read a written instruction of the fMRI task, performed a test trial outside the MRI scanner, and then proceeded with the fMRI task in the MRI scanner.

### 2.3 FMRI task

**FIGURE 1:**
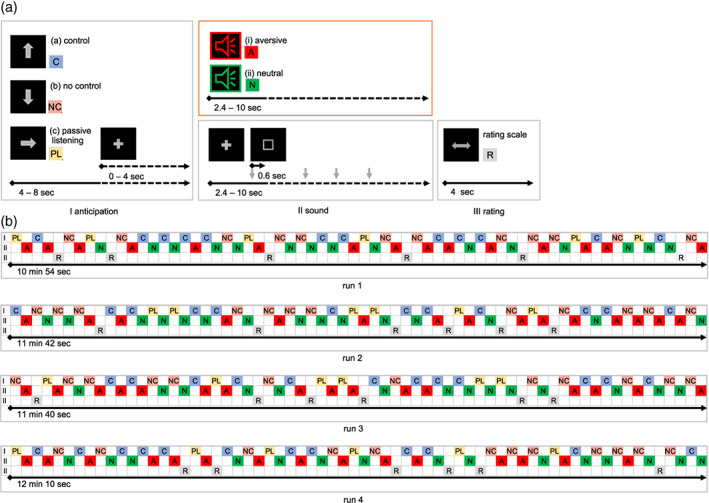
(a) Illustration of one trial including an anticipation (I), sound (II), and rating (III) phase. (b) Illustration of the sequence of (I), (II), and (III) during each of the four runs

Visual and auditory stimuli were presented in an event‐related design. The task consisted of four runs with 30 trials each. The design of one trial is illustrated in Figure 1a. Each trial was composed of an anticipation phase (I), a sound phase (II), and, sometimes, a rating phase (III). During the anticipation phase (I) an arrow indicated if participants (A) had an influence over the length of the sound (control), (B) had no influence over the length of the sound (no control), or (C) should passively listen to the sound (passive listening). If (A) or (B) was indicated, participants were instructed to press a button when they saw a rectangle appear during the presentation of the sound. The participants did not have actual control over the length of the presented sound. During the sound phase (II), participants were exposed to either aversive (i) or neutral sounds (ii). During the rating phase (III), participants rated the aversiveness of the sounds on a 10‐point visual analogue scale from 0 (not aversive) to 9 (very aversive). Each trial was followed by a break during which participants viewed a black screen for 5 to 11 s (pseudorandomized duration) followed by a fixation cross for 1 to 3 s (pseudorandomized duration).

Figure 1b illustrates the arrangement of trials during each of the four runs. During the anticipation phases of each run, 12 arrows indicated (A), 12 arrows (B), and 6 arrows (C). The presentation time of the arrows was pseudorandomized to 4 to 8 s. The arrow was followed by a fixation cross. During the sound phases of each run, 30 sounds were presented out of a repertoire of 14 different sounds. Fifteen sounds were of an aversive quality (e.g., metal scratching, screaming in a high pitch, etc.) (i), and 15 sounds were neutral (e.g., a child laughing, bubbling water, etc.) (ii). The duration of the sound was pseudorandomized to 2.4 to 10 s. During the sound, a fixation cross was shown. Additionally, a rectangle was presented for 0.6 s during the sound indicating that participants should press a button. During the rating phases in each run, each combination of (A), (B), (C), and (i) or (ii) was rated once. The visual analog scale, on which the rating was performed, was presented for 4 s

The duration of a run varied between 10 min 54 s and 12 min 10s. The overall duration of the task including all four runs, a sound test, and the acquisition of a structural T1 scan was ~51 min 31 s. Participants performed 2 to 4 runs (14 performed 4 runs, 10 performed 3 runs, 1 performed 2 runs). The variation in the number of performed runs resulted from the individual (e.g., fatigue) and organizational (e.g., limited scanning resources) constraints. In the case of one participant, 2 runs out of 4, and in the case of five participants, 1 run out of 3 or 4 had to be excluded due to head motion >3 mm. In consequence, we included data of 10 participants with 4 runs, 10 with 3 runs, and 5 with 2 runs in our data analyses. We controlled for these different numbers of runs in our statistical model.

### 2.4 FMRI data acquisition

Data acquisition was performed on a Philips Intera 3 T whole‐body MR unit equipped with a 32‐channel Philips SENSE head coil. Functional time series were acquired with a T2*‐weighted, sensitivity‐encoded single‐shot echo‐planar sequence (SENSE‐sshEPI) (Pruessmann, Weiger, Scheidegger, & Boesiger, 1999). The following acquisition parameters were used in the fMRI protocol: echo time = 35 ms, field of view (FOV) = 220 mm x 220 mm x 128 mm, acquisition matrix = 80 x 80, voxel size: 2.75 mm x 2.75 mm x 4 mm, SENSE acceleration factor R = 2.0. Using a mid‐sagittal scout image, 32 contiguous axial slices were placed along the anterior–posterior commissure plane covering the entire brain with a TR of 2000 ms for task‐based fMRI (θ = 80°) and 3,000 ms for rsfMRI (θ = 82°). Slices were collected in ascending order. The first five acquisitions were discarded to eliminate the influence of T1 saturation effects. An anatomical T1‐weighted structural image was also acquired (FOV = 220 x 220 x 135 mm; acquisition matrix = 224 x 187, interpolated to 224 x 224; reconstructed voxel size = 0.98 x 0.98 x 1.5mm^3^, 180 slices).

### 2.5 Task‐based fMRI analyses

Task‐based fMRI data were analyzed using Matlab version 2015a (The MathWorks, Inc. 2015) and SPM 12 (Statistical Parametric Mapping, Welcome Department of Cognitive Neurology, 2014). Functional images were corrected for time differences in slice acquisition and head motion using the slice time and realign functions of SPM12. We performed segmentation and spatial normalization to Montreal Neurological Institute (MNI) space. Images were smoothed using a Gaussian kernel of 8 mm full width at half maximum (FWHM). None of the participants had to be excluded due to signal dropout. One participant had to be excluded post hoc due to excessive head movement (>3 mm, cf. section *Participants*), and, in the case of six participants, we had to exclude single runs of the fMRI task post hoc due to excessive head movement (>3 mm, cf. section *FMRI task*). At the single‐subject level, we computed a general linear model to obtain parameter estimates of event‐related activity at each voxel, for each condition, and each subject, and statistical parametric maps of the *t* statistic resulting from linear contrasts between different conditions. We modeled the conditions anticipation of control, the anticipation of no control, aversive sound, and neutral sound. Six movement parameters extracted from realignment were entered as regressors. We convolved all explanatory variables with the canonical hemodynamic response function. Our contrasts of interest were (a) anticipation of control > anticipation of no control, and (b) aversive sound > neutral sound. Beta‐weight estimates of contrasts were scaled depending on the number of runs performed by the participants. The individual contrast estimates of all participants were entered into a random‐effects model. Within‐group activation was assessed using one‐sample *t* tests. Whole‐brain cluster inference was completed using false discovery rate (FDR) corrected for multiple comparisons with a threshold of *p* = 0.05. The cluster‐forming height threshold was set to *p* = .001 uncorrected.

## 3 RESULTS

**FIGURE 2:**
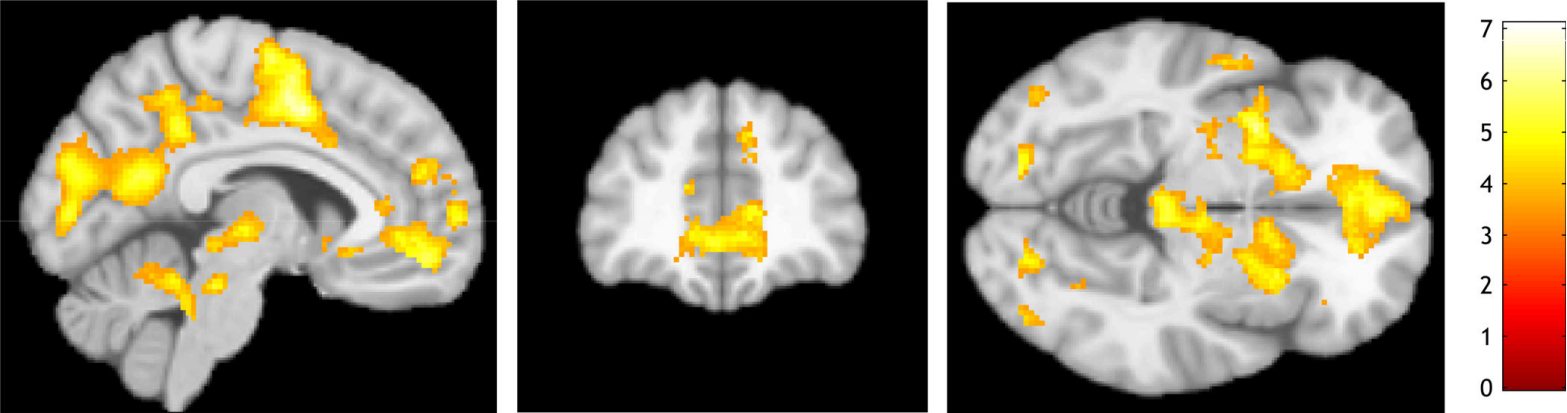
Illustration of results for the contrast anticipation of control > anticipation of no control. Slices are displayed at x = −6, y = 42, z = −6

For the main contrast (a) anticipation of control > anticipation of no control, we found increased neural activation in a large prefrontal cluster comprising the vmPFC and rACC and several reward relevant regions such as the left ventral striatum (cluster extending into the putamen), brainstem—a region consistent with the ventral tegmental area (VTA)—, and MCC (cluster extending into the primary somatosensory cortex) (cf. Table 1 and Figure 2). We also observed increased neural activation in a large cluster comprising the posterior cingulate cortex (PCC) and precuneus, and in smaller clusters in the right posterior insula, left fusiform gyrus, and right inferior occipital gyrus.

**TABLE 1:** Results of whole‐brain analysis for the contrast anticipation of control > anticipation of no control

Brain region	Hemi‐sphere	BA	Coordinates			Cluster size	Cluster *p*(FDR‐corr)
			x	y	z		
vmPFC/rACC	R/L	32, 10	4	48	0	2,158	<.001
PCC/precuneus	R/L	31	20	−60	18	7,422	<.001
Ventral striatum/putamen	L	34	−30	6	−8	1763	<.001
Brainstem (VTA)	R/L		−2	−30	−22	1997	<.001
MCC/ primary somatosensory cortex	L	3	−24	−28	56	241	<.05
Posterior insula	R	13	44	−28	20	161	<.05
Fusiform gyrus	L	37	−34	−48	−18	237	<.05
Inferior occipital gyrus	R	19	42	−80	−2	203	<.05

Abbreviations: BA, Brodman Area; FDR, false discovery rate; L, left; MCC, midcingulate cortex; PCC, posterior cingulate cortex; R, right; rACC, rostral anterior cingulate cortex; vmPFC, ventromedial prefrontal cortex; VTA, ventral tegmental area.

**FIGURE 3:**
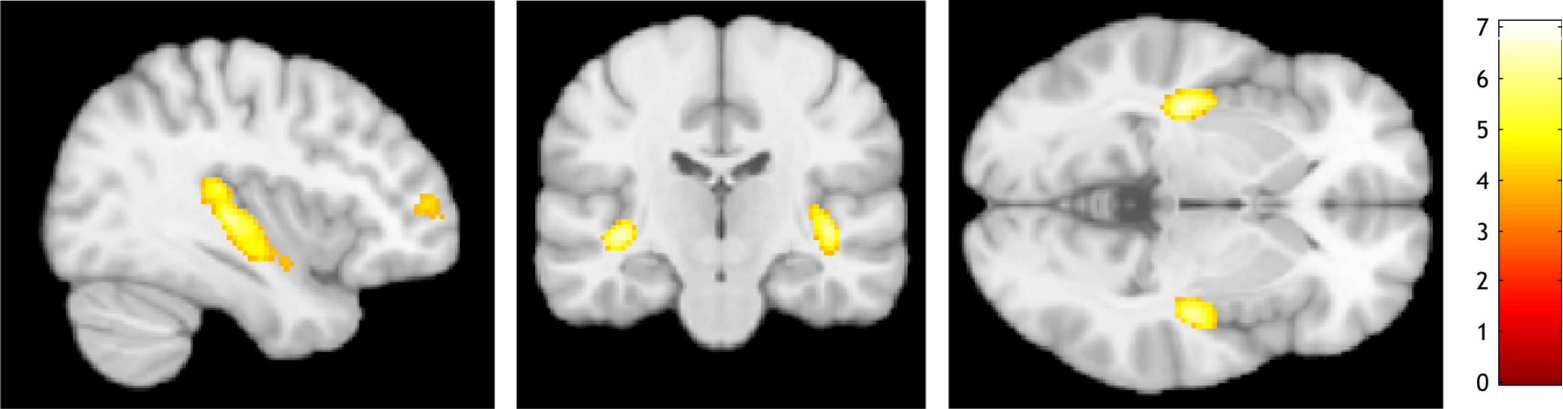
Illustration of results for the contrast aversive sound > neutral sound. Slices are displayed at x = 38, y = −20, z = −3

In the contrast (b) aversive sound > neutral sound, we observed increased neural activation in the bilateral posterior insula (cf. Table 2 and Figure 3). These relatively large clusters of neural activation extended into the superior part of the temporal lobe including the auditory cortex (Brodman Areas 41, 22).

**TABLE 2:** Results of whole‐brain analysis for the contrast aversive sound > neutral sound

Brain region	Hemis‐phere	BA	Coordinates			Cluster size	Cluster *p*(FDR‐corr)
			x	y	z		
Posterior insula/ superior temporal gyrus	R	13, 41, 22	34	−28	10	638	<.001
Posterior insula/ superior temporal gyrus	L	13, 41, 22	−40	−12	−4	843	<.001

Abbreviations: BA, Brodman area; FDR, false discovery rate; L, left; R, right.

## 4 DISCUSSION

The aim of this study was to explore neural correlates of (1) the anticipation of control over aversive auditory stimulation and (2) the processing of aversive auditory stimulation in a newly developed fMRI task with healthy participants. We found that (1) anticipating control in contrast to no control over aversion was accompanied by a higher neural activation in a large medial prefrontal cluster comprising the vmPFC and rACC, several other regions of the reward system such as the left ventral striatum and the brainstem (in a region consistent with the VTA), and the PCC. We observed (2) increased neural activation in the bilateral posterior insula during the processing of aversive compared to neutral auditory stimulation. Our findings suggest the implication of medial prefrontal areas and additional brain regions important for the processing of rewards and self‐relevant stimuli in control anticipation in humans.

### 4.1 Neural correlates of the anticipation of control over aversive auditory stimulation

Our findings mostly align with our initial hypotheses expecting increased neural activation in regions implicated in the anticipation of control over aversion and rewards such as the vmPFC, rACC, and ventral striatum. However, we did not find the expected modulations of neural activation in the amygdala and anterior insula.

These results provide evidence for the implication of the medial prefrontal cortex in the anticipation of control over aversion in humans: In our fMRI task, anticipating control was associated with increased neural activation in a large medial prefrontal cluster including the vmPFC and rACC. Based on findings mostly in rodents, Maier and Seligman (2016) postulated that the vmPFC takes a crucial role in the expectation to control aversive stimulation. Evidence from neuroimaging studies in humans had remained inconclusive so far (Kerr et al., 2012; Wood et al., 2015). Shenhav, Cohen, and Botvinick (2016) highlight the implication of the rACC in both cognitive control and reward processing. They proposed that the rACC is crucial for allocating control resources dependent on the expected benefit for the organism, a process likely mobilized by control anticipation in our task.

Additionally, we observed increased neural activation in the ventral striatum and the brainstem (in a region consistent with the VTA) during control anticipation. Together with the vmPFC and rACC, the ventral striatum and the VTA have been consistently associated with reward processing and its anticipation (Gu et al., 2019; Smith & Delgado, 2015; Wilson et al., 2018). These regions are part of the mesocorticolimbic dopamine pathways, which originate in the VTA and project into the ventral striatum and prefrontal cortex (Arias‐Carrión, Stamelou, Murillo‐Rodríguez, Menéndez‐González, & Pöppel, 2010). In line with Ly et al. (2019), we argue that this implication of reward‐processing regions may be linked to the affective and motivational aspects of control anticipation. In fact, anticipating control over aversive stimulation may have an inherent appetitive value for the study participants in our fMRI task. Several other studies have demonstrated that exercising control is inherently rewarding (for a review, see Leotti, Iyengar, & Ochsner, 2010). The implication of striatal regions in these processes is well‐established. For instance, Tricomi, Delgado, and Fiez (2004) showed that having a choice in a reward or punishment entailing oddball paradigm was associated with increased striatal activation. Similarly, Coricelli et al. (2005) observed higher neural activation in the ventral striatum during a gambling task only when participants had a choice. Different parts of the striatum seem to be functionally related to different characteristics of choices (representations of the value of freedom and opportunity to choose) (Fujiwara et al., 2013). Leotti and Delgado (2011) used a monetary reward task where cues indicated choice, potential choice, or no choice. Similar to our findings, the authors showed that expecting a choice was associated with increased neural activation in the ventral striatum and the dorsal ACC. In a subsequent study, Leotti and Delgado (2014) explored if neural circuits involved in choice opportunity depended on the valence of potential outcomes. They demonstrated that choice entailed greater ventral striatal activation when processing losses but not when gains were involved in the experimental procedure. This indicates that the affective and motivational aspects of control anticipation are context dependent. In sum, our findings of increased neural activation in the vmPFC, rACC, ventral striatum, and VTA fit well into the existing literature that emphasizes the implication of reward‐processing regions in control anticipation. However, it should be noted that our results do not necessarily have to reflect reward processing but might also be (partially) described by Pavlovian transfer in the aversive domain. For instance, Geurts, Huys, den Ouden, and Cools (2013) found aversive Pavlovian transfer to be reflected in neural activation within the amygdala and the ventral striatum. Consequently, our findings within the ventral striatum could also be explained by aversive Pavlovian transfer.

We also observed increased MCC activation during control anticipation in a large cluster that extended into the primary somatosensory cortex. The MCC has also been associated with the anticipation of rewards and aversion (Andrzejewski et al., 2019; Wilson et al., 2018). Andrzejewski et al. (2019) pointed out that the MCC may be implicated in the initiation or modulation of the autonomic response to a threat. Vogt (2016), however, described a broader array of behavioral and cognitive processes mediated by the MCC. He clarified the differential functional and structural properties of the anterior and posterior parts of the MCC. The anterior MCC has high dopaminergic afferents and is implicated in action‐reinforcement association and approach or avoidance decision‐making. We assume that our task mobilized these types of processes more strongly when participants anticipated control compared to no control. However, this interpretation is only one among many possible ones as the MCC is likely implicated in many cognitive and behavioral processes (Vogt, 2016).

Furthermore, control anticipation was associated with increased neural activation in a large cluster comprising the PCC and precuneus. Together with the medial prefrontal cortex, these are the core nodes of the default mode network (DMN). The DMN is typically deactivated during tasks and underpins mind wandering and thoughts related to one's self (Raichle, 2015). It has also been demonstrated that these medial core regions of the DMN underpin self‐relevant decision‐making (Andrews‐Hanna, Reidler, Sepulcre, Poulin, & Buckner, 2010). Anticipating control in contrast to anticipating no control may mobilize an experience of agency that is underpinned by similar neural pathways as previously observed for self‐relevant decision‐making. In this line of thought, it is noteworthy that recent meta‐analyses demonstrated that neuroimaging studies of reward and self‐referential processes found neural activation in both differential and common brain regions (Frewen et al., 2020). Common brain regions were especially identified in the medial prefrontal cortex including the vmPFC and rACC where we observed increased neural activation during control anticipation.

Overall, these findings suggest that control anticipation is mediated by medial prefrontal regions that have been implicated in control anticipation in rodents and the allocation of control resources in humans as well as reward processing. Control anticipation was further associated with increased neural activation in additional regions important for reward anticipation such as the ventral striatum and brainstem (VTA). Finally, control anticipation led to increased neural activation in medial brain regions of the DMN that typically activate during the processing of self‐relevant stimuli. It is important to address that most of these brain regions underpin complex cognitive and affective functions. Additional and divergent functional interpretations of their activation during control anticipation are thus possible.

### 4.2 Neural correlates of the processing of aversive auditory stimulation

We observed enhanced neural activation in the bilateral posterior insula during the processing of aversive compared to neutral sounds. These results did not match our initial hypotheses concerning the neural activation during the processing of aversive compared to neutral sounds. We had expected increased neural activation in the bilateral anterior insula, the rACC, bilateral ventrolateral orbitofrontal cortex, and bilateral amygdala, yet observed no modulation of neural activation in these brain regions. Concerning insular activation, we had expected an increase in its anterior and not posterior part as this has been a relatively consistent finding in the context of experiencing pain. Interestingly, some studies also suggest that the posterior part of the insula may specifically play a role in pain processing. For instance, Lamm, Decety, and Singer (2011) suggested a posterior‐to‐anterior gradient within the insula with posterior parts of the insula being only activated by pain directly perceived by oneself while the anterior insula covering both self‐perceived pain and pain inflicted in others. Consequently, our findings in the posterior insula may reflect pain processing. Nonetheless, it is important to note the absence of activation in other brain areas, which does not match the commonly observed results of previous studies on pain (Hayes & Northoff, 2012; Lamm et al., 2011). We speculate that this inconsistency may link to the intensity of the aversive experience induced by the auditory stimuli employed in our task. Possibly, these auditory stimuli were experienced as less aversive compared with aversive stimulation used in previous studies such as for example, inflicting tactile pain. However, this speculation is contradicted by observations of two meta‐analyses on the anticipation and processing of aversion (Andrzejewski et al., 2019; Hayes & Northoff, 2011), which had found no difference in neural activation depending on the modality of stimuli used.

The clusters of neural activation in the posterior insula were large and extended into parts of the superior temporal lobe including the auditory cortex. This finding aligns with previous observations demonstrating that negatively valanced auditory stimuli are associated with increased neural activation in the auditory cortex (Kumar, von Kriegstein, Friston, & Griffiths, 2012; Viinikainen, Kätsyri, & Sams, 2012). Kumar et al. (2012) proposed that the auditory cortex first processes salient auditory stimuli before an emotional response can be coordinated in other brain areas. It is also interesting to note that an fMRI study on the neural underpinnings of mourning found neural activation in bilateral superior temporal clusters similarly located to the ones we observed here (Labek et al., 2017). Labek et al. (2017) argued that these temporal regions are part of a posterior network important for the appraisal of pain and especially its sensory experience, which is consistent with our findings.

### 4.3 Limitations

Our study is not without limitations. First, our fMRI task does not allow to differentiate between control anticipation over aversion or rewards. Its focus was to explore neural correlates of the anticipation of control over aversive auditory stimuli only. Our fMRI task could have been complemented by a condition where the anticipation of control related to rewarding stimuli. This would have enabled us to differentiate between control anticipation over aversive and rewarding stimuli. Second, a substantial limitation of the analyses reported here is that we did not account for the subjective experience of control anticipation and aversion. We did not consider any subjective ratings of these aspects. Third, participants did not have actual control over the lengths of the sounds that were presented. We do not know if this caused feelings of frustration or confounded the difference of the control versus no control condition during the course of the experiment. Fourth, our sample size limits the power of our analyses. Therefore, our results need to be interpreted with caution and necessitate replication in a larger sample. Fifth, our sample is imbalanced in terms of gender (17 females, 8 males). We did not explore the effects of gender due to our limited sample size.

### 4.4 Future directions

Anticipating control over future events is highly relevant for our mental health and the anticipation of aversion is important to avoid harmful experiences. Aberrant control anticipation and altered aversion processing is an important component of pathological pathways in several mental disorders such as depression, anxiety disorders, schizophrenia, and addiction (Gallagher et al., 2014; Hayes & Northoff, 2011; Liu et al., 2015). It will thus be interesting to explore the neural correlates of such altered processes in psychopathology. Our fMRI task can be a useful instrument for such an endeavor.

## CONFLICT OF INTERESTS

The authors declare no conflicts of interest.

## Data Availability

The data that support the findings of this study are available on request from the corresponding author. The data are not publicly available due to privacy or ethical restrictions.
